# Malpighian Tubule Cells in Overwintering Cave Crickets *Troglophilus cavicola* (Kollar, 1833) and *T*. *neglectus* Krauss, 1879 (Rhaphidophoridae, Ensifera)

**DOI:** 10.1371/journal.pone.0158598

**Published:** 2016-07-05

**Authors:** Saška Lipovšek, Tone Novak, Franc Janžekovič, Nina Weiland, Gerd Leitinger

**Affiliations:** 1 Faculty of Medicine, University of Maribor, Maribor, Slovenia; 2 Department of Biology, Faculty of Natural Sciences and Mathematics, University of Maribor, Maribor, Slovenia; 3 Faculty of Chemistry and Chemical Engineering, University of Maribor, Maribor, Slovenia; 4 Institute of Cell Biology, Histology and Embryology, Research Unit Electron Microscopic Techniques, Medical University of Graz, Graz, Austria; USDA-ARS, UNITED STATES

## Abstract

During winter, cave cricket larvae undergo dormancy in subterranean habitats; this dormancy is termed diapause in second year *Troglophilus cavicola* larvae because they mature during this time, and termed quiescence in *T*. *neglectus*, because they mature after dormancy. Here we used electron microscopy to analyze ultrastructural changes in the epithelial cells in the Malpighian tubules (MTs) of *T*. *cavicola* during diapause, in order to compare them with previous findings on *T*. *neglectus*. Moreover, the autophagosomes were studied with immunofluorescence microscopy in both species. Although the basic ultrastructure of the cells was similar, specific differences appeared during overwintering. During this natural starvation period, the nucleus, rER, the Golgi apparatus and mitochondria did not show structural changes, and the spherites were exploited. The abundances of autophagic structures in both species increased during overwintering. At the beginning of overwintering, in both species and sexes, the rates of cells with autophagic structures (phagophores, autophagosomes, autolysosomes and residual bodies) were low, while their rates increased gradually towards the end of overwintering. Between sexes, in *T*. *cavicola* significant differences were found in the autophagosome abundances in the middle and at the end, and in *T*. *neglectus* at the end of overwintering. Females showed higher rates of autophagic cells than males, and these were more abundant in *T*. *cavicola*. Thus, autophagic processes in the MT epithelial cells induced by starvation are mostly parallel in diapausing *T*. *cavicola* and quiescent *T*. *neglectus*, but more intensive in diapausing females.

## Introduction

The rhaphidophoridan cave crickets (Rhaphidophoridae, Ensiphera) are scotophilic, nocturnal Ensifera inhabiting tropical, subtropical and temperate regions [1–6]. Many of them seek for shelters in caves and other subterranean habitats, or they overwinter there, and a few are adapted for permanent living in subterranenan habitats. In Europe the family is represented by the genera *Dolichopoda* and *Troglophilus*, with about 70 species described to date [7, 8]. Only the genus *Troglophilus* Krauss, 1879 extends its range to central Europe, with two of the 19 species described until recently: *T*. *neglectus* Krauss, 1879 and *T*. *cavicola* (Kollar, 1833) [8–17]. They are among the most numerous invertebrates, with the largest fresh and dry mass bulk among invertebrates overwintering in central European caves [18].

During overwintering, the larvae of both cave cricket species do not feed, as is typical in insects during dormancy, which is a state of temporarily reduced metabolic activity during unfavorable conditions [19]. The larvae of *T*. *cavicola* hatch in May and live in epigean habitats until the late autumn. The younger larvae enter hypogean habitats (in the following text: caves) and overwinter there in quiescence from November until March. Then they live outside until the next autumn when they return, as older larvae, to the caves for the second time. During the second overwintering, they carry out a diapause reaching their maturity, mate and lay eggs in March, and die by June [10, 13, 20, 21]. These conditions are similar in *T*. *neglectus*, but these enter caves 2–4 weeks later and leave them 2–4 weeks earlier, carrying out a quiescence during both overwintering periods [11, 20, 21]. Older *T*. *neglectus* larvae mature, mate and lay eggs in late June or July. Thus, both *Troglophilus* species are convenient subjects for studying the influences of stress conditions caused by natural starvation.

The Malpighian tubules (MTs) are the most important excretory and osmoregulatory organs in many myriapods, arachnids and insects [22]. As for their function, the MTs can be compared with the vertebrate kidney tubules, since both transport organic solutes, break down and remove or excrete toxic substances, and maintain ionic balance and immune defenses [23, 24]. MTs actively and passively absorb water and ions from the hemolymph inside the coelom and convert waste metabolites into urine compounds, which move towards the hindgut. The rectum performs osmoregulation within the body by reuse of water and ions [25]. MTs are blind-end tubular organs which extend from the midgut–hindgut junction into the hemocoel. The MT is composed by a single-layered epithelium and individual muscle cells, which are thought to mix the contents of the MT and displace the MT in the haemolymph [22]. The plasma membrane of the epithelial cell is characterized by numerous apical microvilli and numerous prominent infoldings in its basal part. These cells transport fluid and solutes and have a storage role [26].

Autophagy is an evolutionarily conserved self-digestive process in eukaryotic cells adapted to nutrient starvation [27, 28]. It is a common response in starving invertebrates providing cells with needed nutrients [29–32]. The LC3 protein is specifically associated with autophagosomes; consequently, an increase in the amount of the LC3 protein correlates well with an increased number of autophagosomes [33].

Because the two closely related cave cricket species, *Troglophilus cavicola* and *T*. *neglectus*, implement different overwintering strategies during the second year, they are of special interest for comparison to find eventual differences between diapause and quiescence on the cell level. During overwintering, cave crickets do not feed.

Changes in the following two organs are best representative for understanding this natural starvation period. The fat body is the central storage depot for excess nutrients [34] intensively supplying energy for maintaining overwintering insects alive. Besides, MTs show the final products of the cell homeostasis processes during this period. They maintain constant internal environment through the elimination or segregation of waste substances [35, 36]. The MT epithelial cells are appropriate for this study because they contain the final compounds prior to excretion. Consequently, these two organs were of major interest in our research. The fat body had previously been studied in both *T*. *cavicola* and *T*. *neglectus* [37, 38] and the MT epithelial cells had been researched in *T*. *neglectus* [21]. In this study we complete the previous research on the MTs of *T*. *cavicola*. We asked how epithelial MT cells respond in the diapausing *T*. *cavicola*. Our aim was to analyze the ultrastructure of these cells, and to estimate the number of autophagosomes within these cells in both species during overwintering. For this purpose, autophagic structures in the MT epithelial cells were examined in both species. It was hypothesized that the abundance of the autophagic structures will increase from the beginning until the end of overwintering in both species. A higher number of autophagosomes was expected in diapausing *T*. *cavicola* with respect to quiescent *T*. *neglectus*. Finally, we summarize the findings in the fat body and MT epithelial cells in diapausing *T*. *cavic*ola vs. quiescent *T*. *neglectus*.

## Material and Methods

### Material

The cave crickets *T*. *cavicola* and *T*. *neglectus* were collected from four caves in central northern Slovenia (locality centroid 46°24´55˝ N, 15°10´31˝ E, altitudes 600–740 m), in the territory of the Republic Slovenia with a state permission to one of the co-authors for such researches. These species are neither endangered nor protected. In previous studies on MTs and the fat body it was found that individual differences in the MT cells structure are very limited, and their ultrastructures are comparable [21, 37]. In the pre-study on 30 individuals of each species and sex we found a unified ultrastructure within the cells. Therefore, for the current study, seven *T*. *cavicola* and *T*. *neglectus* of each sex were analyzed microscopically just before the beginning (November) (in the following text: beginning), in the middle (January) and at the end (March) of overwintering. In each individual 25 MTs were analyzed by light microscopy and transmission electron microscopy (TEM) and 25 MTs by immunofluorescence microscopy (IFM). In each time frame/species/sex at least 300 cells were examined, and representative images are shown here.

### Methods

The middle section of MTs, embracing about 90% of a MT, with typical epithelial cells was investigated. The structure of the MTs epithelial cells in *T*. *cavicola* was examined by light microscopy and transmission electron microscopy (TEM), as had been done in *T*. *neglectus* [21], and both species were examined by immunofluorescence microscopy (IFM) for LC3 localization in autophagosomes at the three time frames during overwintering. We used the marker LC3B primary antibody, commonly used in studies of autophagy [33].

### Light and transmission electron microscopy (TEM)

The MTs were fixed in 2.45% glutaraldehyde and 2.45% paraformaldehyde in a 0.1 M sodium cacodylate buffer (pH 7.4) at room temperature for 2 hrs and at 4°C for 12 hrs. The tissue was washed in a 0.1 M sodium cacodylate buffer (pH 7.4) at room temperature for 3 hrs and postfixed with 2% OsO_4_ at room temperature for 2 hrs. The samples were dehydrated in a graded series of ethanol (50%, 70%, 90%, 96%, 100%, each for 30 minutes at room temperature) and embedded in TAAB epoxy resin (Agar Scientific Ltd., Essex, England). For light microscopy, semi-thin sections (5 μm) were used stained with 0.5% toluidine blue in aqueous solution. For TEM, ultra-thin sections (70–75 nm) of the MTs were transferred onto copper grids, stained with uranyl acetate and lead citrate and analyzed with a Zeiss EM 902 transmission electron microscope.

Ultra-thin sections have been used in order to estimate the ratios of epithelial cells containing autophagic structures−phagophores, autophagosomes, autolysosomes and residual bodies−to the total number of cells in the sample. For this measurement, 100 MT epithelial cells for each time frame and each sex were randomly selected. Random counting was carried out at 3000x magnification.

### Immunofluorescence microscopy (IFM) for LC3 localization

The MTs were dissected in phosphate buffered saline (PBS) and fixed in 3.7% formaldehyde (in PBS as diluent) for 20 min at room temperature. The tissue was rinsed with PBS-Tx (PBS containing 0.2% Triton X 100) three times for 5 min each, and permeabilized in 0.2% Triton X 100 in PBS for 20 min at room temperature. To block possible non-specific binding, the tissue was incubated in BlockAid^™^ Blocking Solution (Molecular Probes) for 1 hour at room temperature. Afterwards, the samples were rinsed with PBS-Tx three times for 5 min each and treated with primary antibodies LC3B (rabbit polyclonal antibody, Molecular Probes, Cat. Nr. L10382) diluted in PBS-Tx (1:250) for 14 hours at 4°C. The tissue was washed with PBS-Tx three times for 5 min each, incubated with secondary goat anti-rabbit IgG antibodies (Molecular Probes) diluted in PBS-Tx (1:100) for 2 hours at room temperature, and washed with PBS-Tx three times for 5 min each. The epithelial cells of the MTs were examined under an inverted confocal laser scanning fluorescence microscope Leica TCS SP5 II. The samples were excited with Argon laser line at 488 nm. Quantification of autophagosomes in the MT cells was performed on unitary image areas [39] in November, January and March. We used the images of immunofluorescence microscopy acquired with a 40x oil immersion objective (NA 1.30) at 800x magnification after applying LC3 to localize the autophagosomes. To control for potential false-positive signals from the immunofluorescence analyses, the pieces of the MTs were incubated with secondary antibodies without the primary antibody.

### Statistical analysis

The abundances of autophagosomes in the MTs epithelial cells were tested for normality of distribution using the Kolmogorov-Smirnov test. The distribution of autolysosome abundance was not normal in November, but normal in January and March. Therefore, the differences between the abundances of autophagosomes at different time frames, between the sexes and between the two species were tested using the The Mann-Whitney U test, which does not require normal distributions.

## Results

### Malpighian tubules

**Fig 1:**
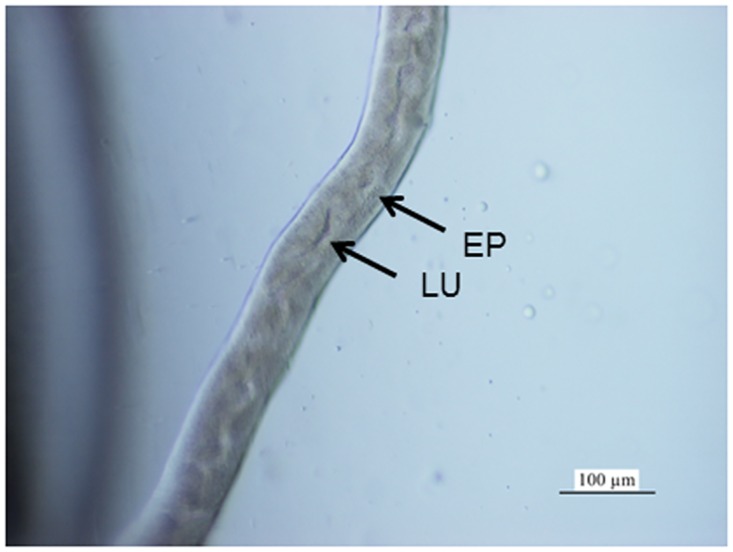
A part of the Malpighian tubule (MT) of *T*. *cavicola* at the beginning of overwintering. Light microsccopy allows to see the general appearance of a MT composed of the epithelium (EP), and the lumen (LU).

**Fig 2:**
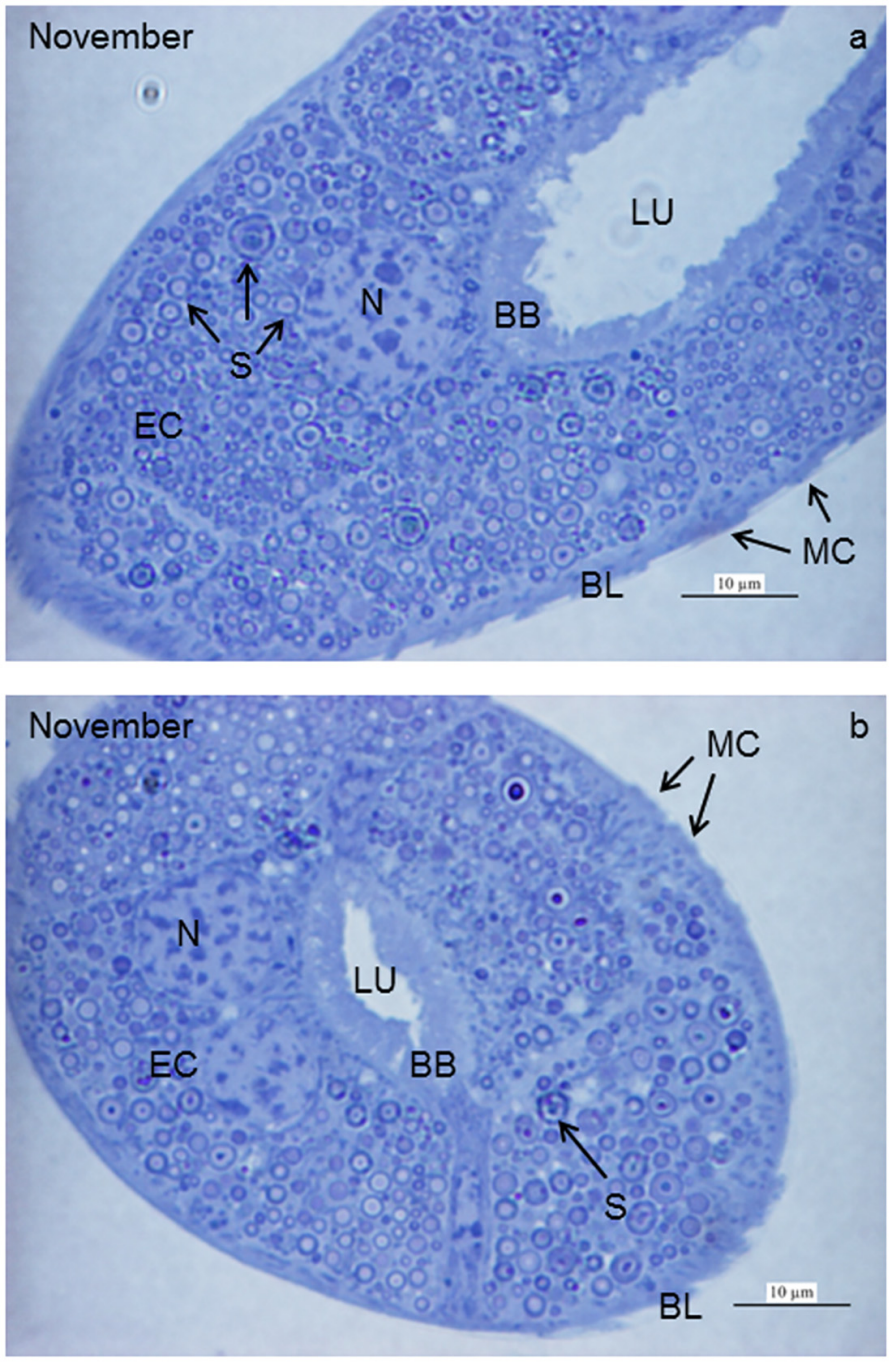
Semithin cross-section of the Malpighian tubule (MT) in *T*. *cavicola* (a) and *T*. *neglectus* (b) at the beginning of overwintering. The epithelium is composed of columnar epithelial cells (EC) and underlying basal lamina (BL). The apical plasma membrane of the ECs is differentiated into the brush border (BB). The cytoplasm is filled with numerous spherites (S). LU, lumen of the MT; MC, individual muscle cells; N, nucleus of the epithelial cell. Note that this figure shows an oblique transection of the MT.

About 50–60 MTs (Fig 1) extended from the midgut-hindgut junction in *T*. *cavicola* (Fig 2a) and *T*. *neglectus* (Fig 2b). These were unevenly scattered in the haemocoel between the digestive tract and the body wall. In the MTs, three morphologically distinct regions could be distinguished: the proximal, the middle and the distal region. The middle part of the MT occupied its major portion, while the proximal and the distal regions each measured less than 10% of the MT length. The outer diameters of the proximal and the distal regions were about 50 μm and that of the middle portion 60–80 μm. The MTs were composed of the epithelium and individual muscle cells attached to the outer MT surface. The epithelium of the middle part of the MT consisted of columnar cells and the basal lamina (Fig 2a and 2b).

The general structure of the MT epithelial cells was comparable throughout overwintering, but showed some changes in the structure of spherites, and in the presence of autophagic structures. In January and March, various autophagic structures were present in the cells.

### Beginning of overwintering

**Fig 3:**
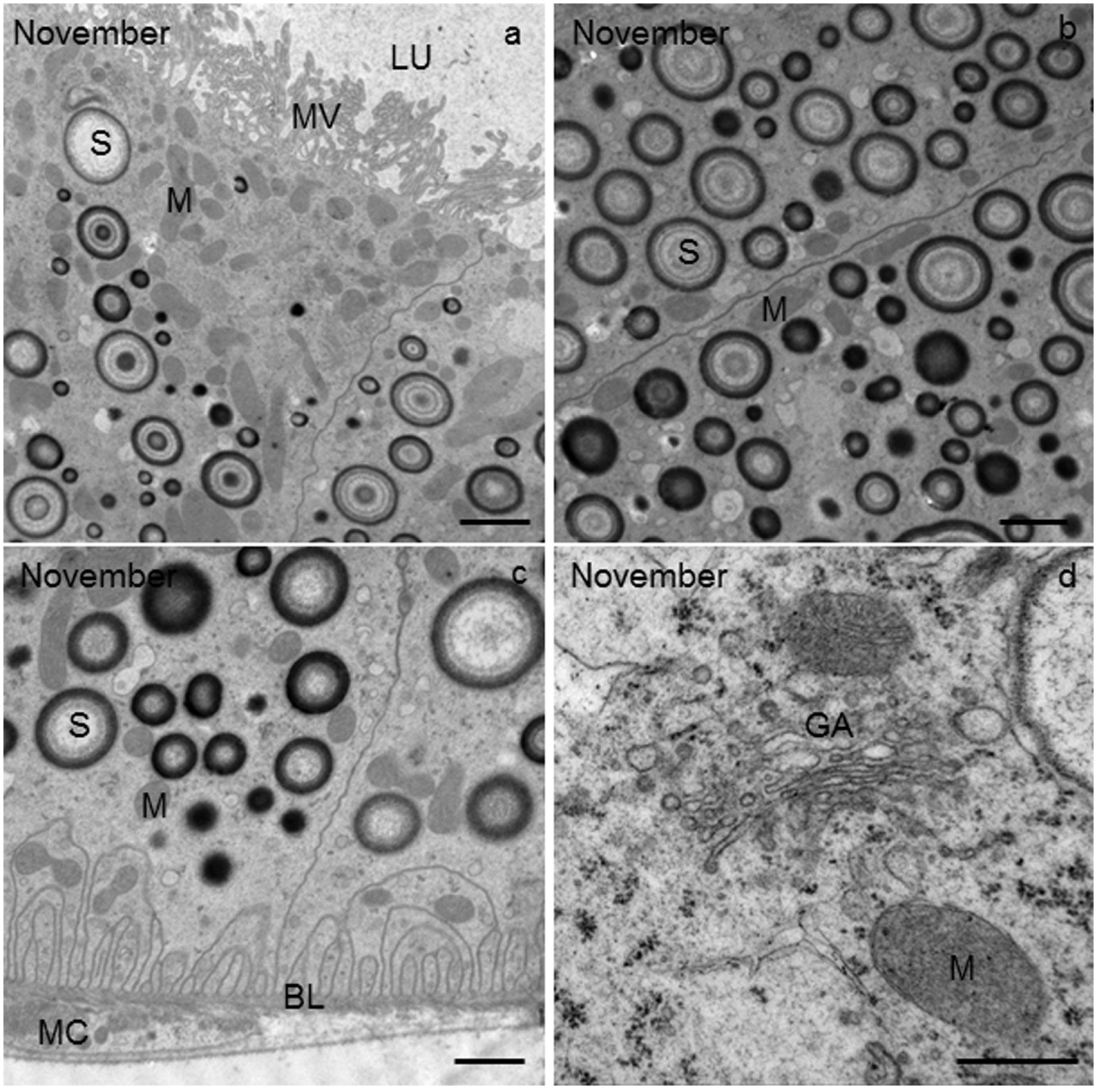
Ultrathin cross-section of the Malpighian tubule (MT) in *T*. *cavicola* at the beginning of overwintering. **(a)** The apical part of the epithelial cell with numerous mitochondria (M) and spherites (S). The apical plasma membrane is differentiated into microvilli of up to 5 μm long (MV). LU, lumen of the MT. **(b)** The perinuclear cytoplasm containing many mitochondria (M) and spherites (S). **(c)** The basal part of the epithelial cell with spherites (S) and mitochondria (M). The basal plasma membrane with typical multiple infoldings and a few mitochondria. Muscle cell (MC) beneath the epithelium of the MT. BL, basal lamina. **(d)** Golgi apparatus (GA) and mitochondria (M) in the perinuclear region of the epithelial cell. Scale bar: 2 μm (a, b, c) and 500 nm (d).

**Fig 4:**
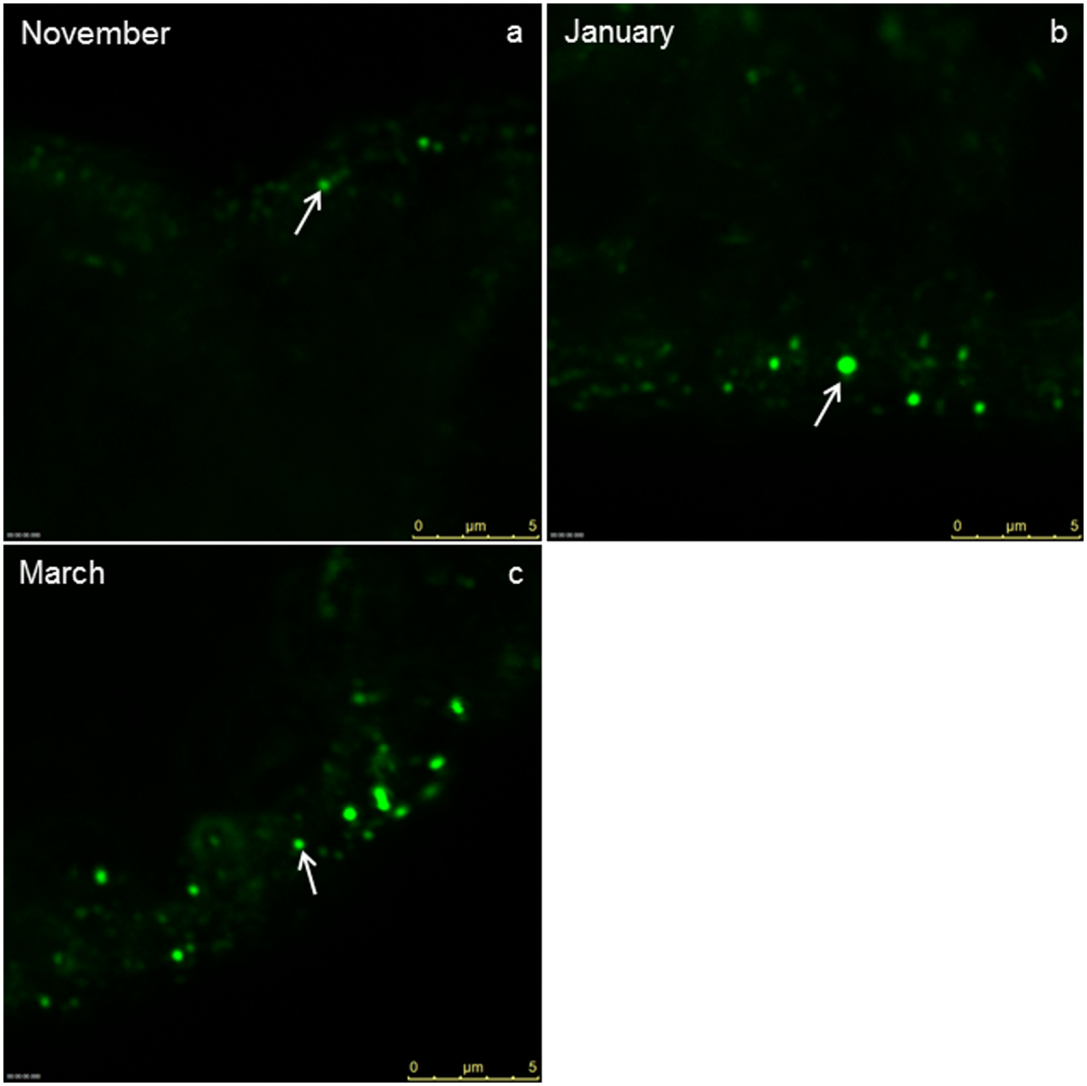
IFM-Visualization of autophagosomes labelled with antiserum against LC3. Immunofluorescent images show autophagosomes (arrows) in the epithelial cells of the Malpighian tubules of *T*. *cavicola* at the beginning of the overwintering (a), in the middle (b) and at the end of overwintering (c).

The apical plasma membrane of the epithelial MT cells of *T*. *cavicola* was differentiated into microvilli of up to 5 μm in length, forming a brush border in the tissue observed by light microscopy (Figs 2 and 3a). The apical and the perinuclear cytoplasm were rich in mitochondria and spherites (Fig 3a). The spherites were composed of various concentric layers of electron-lucent and electron-dense material (Fig 3b). In the basal part of the epithelial cell, numerous mitochondria and spherites were seen (Fig 3c). The multiple infoldings of the basal plasma membrane included a few mitochondria in between (Fig 3c). The epithelium was underlayed by muscle cell (Fig 3c). The perinuclear cytoplasm additionally contained the rER and Golgi apparatus (Fig 3d). With IFM, autophagic structures were rarely observed (Fig 4a, Table 1).

**Table 1:** Rates (in %) of the Malpighian tubule epithelial cell samples containing autophagic structures in *Troglophilus cavicola* and *T*. *neglectus* during overwintering, observed by TEM.

Species	Sex	Time frame of overwintering		
		Beginning	Middle	End
***T*. *cavicola***	Male	7	52	70
	Female	0	64	82
***T*. *neglectus***	Male	7	48	70
	Female	0	55	75

### Middle of overwintering

**Fig 5:**
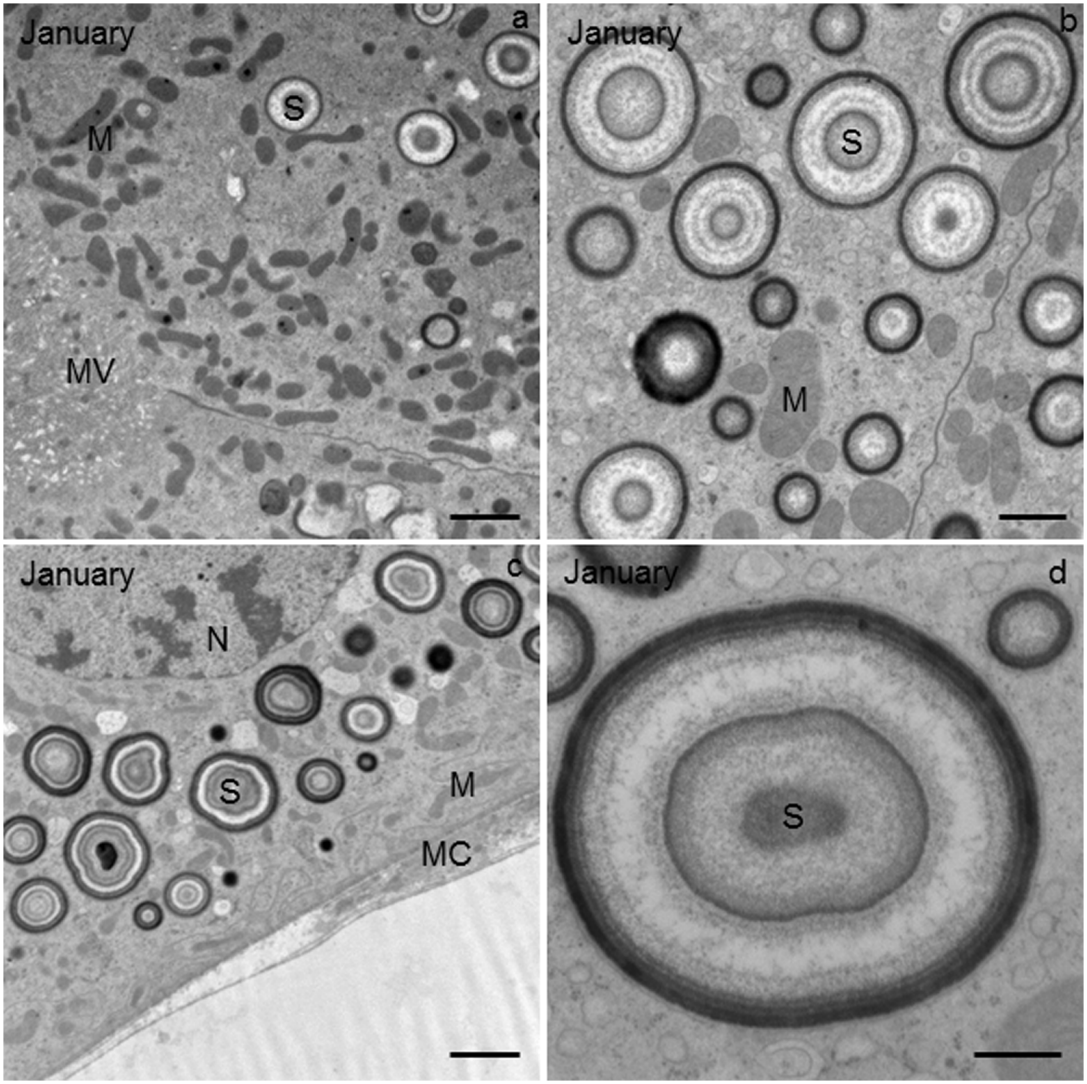
Ultrathin cross-section of the Malpighian tubule (MT) in *T*. *cavicola* in the middle of overwintering. **(a)** Apical part of the cell with numerous mitochondria (M). Spherites (S) containing electron-lucent concentric layers. MV, microvilli. Scale bar: 2 μm. **(b)** Perinuclear region of the epithelial cell with mitochondria (M) and spherites (S). Scale bar: 1 μm. **(c)** The basal part of the epithelial cell with the nucleus (N), spherites (S) and mitochondria (M). MC, muscle cell. Scale bar: 2 μm. **(d)** A spherite (S) composed of electron-lucent and electron-dense concentric layers. Scale bar: 500 nm.

**Fig 6:**
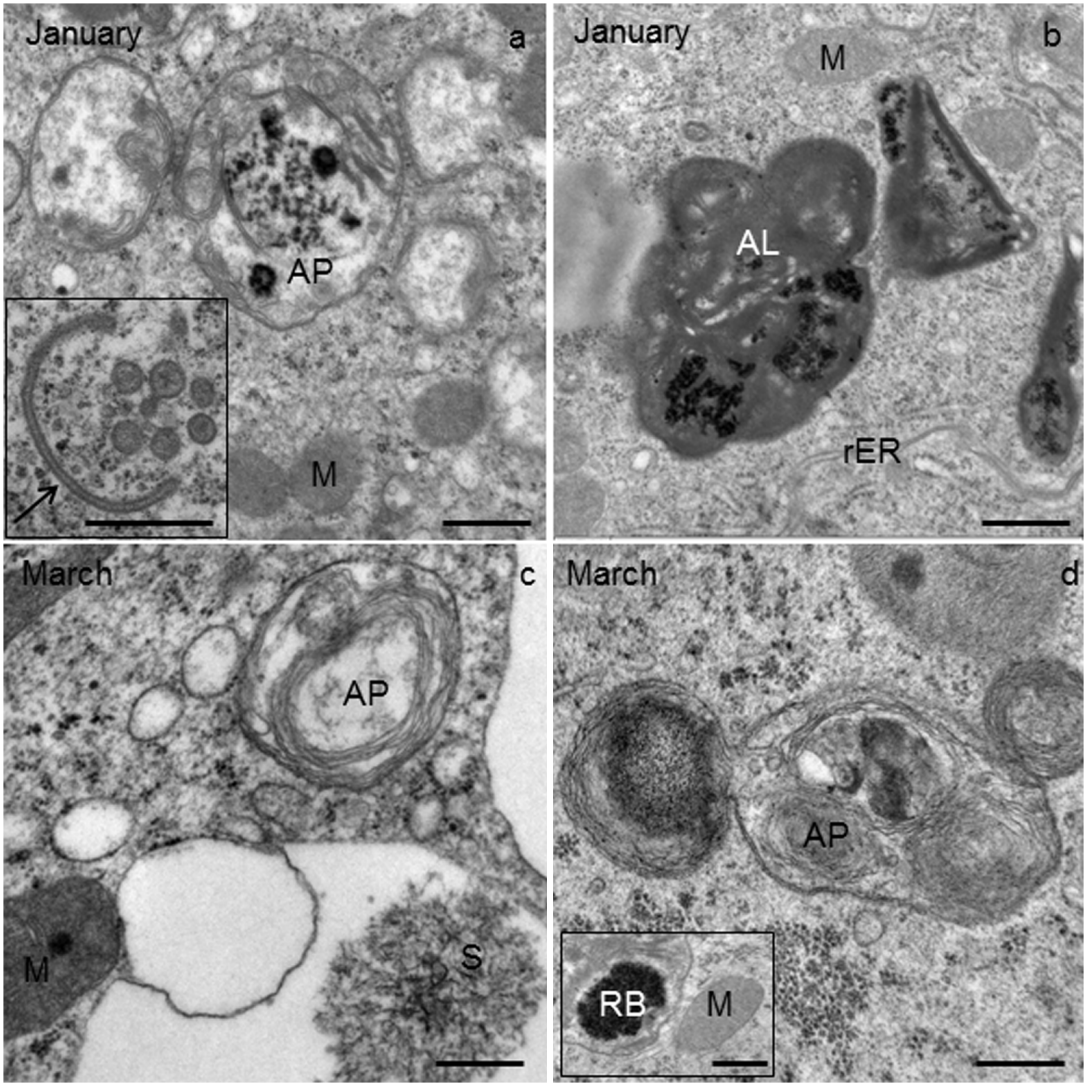
Ultrathin cross-section of the Malpighian tubule (MT) in *T*. *cavicola* in the middle (a, b) and at the end of overwintering (c, d). **(a)** Autophagosome (AP) composed of granular electron-dense material and fragments of the membranes. Scale bar: 500 nm. Inset: the phagophore (arrow). Scale bar: 500 nm. **(b)** Autolysosome (AL). Scale bar: 500 nm. **(c)** Autophagosome (AP), a spherite (S) and a mitochondrium (M) in the perinuclear cytoplasm. Scale bar: 500 nm. **(d)** Autophagosome (AP) containing material of variable electron density. Scale bar: 500 nm. Inset: a residual body (RB) and a mitochondrium (M). Scale bar: 500 nm.

The compartments such as rER, Golgi apparatus, mitochondria apparently did not change in *T*. *cavicola*. In the perinuclear cytoplasm, spherites were as abundant as at the beginning of overwintering, but most of them contained fewer electron-dense and more electron-lucent layers than at the beginning of overwintering (compare Fig 5a–5d, with Fig 3b). By this time various autophagic structures−the phagophores, autophagosomes, autolysosomes and residual bodies−were present in some cells. The phagophores (Fig 6a, inset) were composed of short fragments of the membrane partly engulfing the cytoplasm. Autophagosomes (Fig 6a), were also detectable using IMF (Fig 4b). These contained material of varying electron density. The autophagosomes were composed of electron-dense granules and electron-lucent material enveloped by a double-membrane (Fig 6a). The autolysosomes (Fig 6b), which can be recognized by electron-dense contents enveloped by a single membrane, were also found, showing the disintegration processes of the engulfed material. Residual bodies were recognized by an undegraded, electron-dense remnant material enveloped by a single membrane.

### End of overwintering

**Fig 7:**
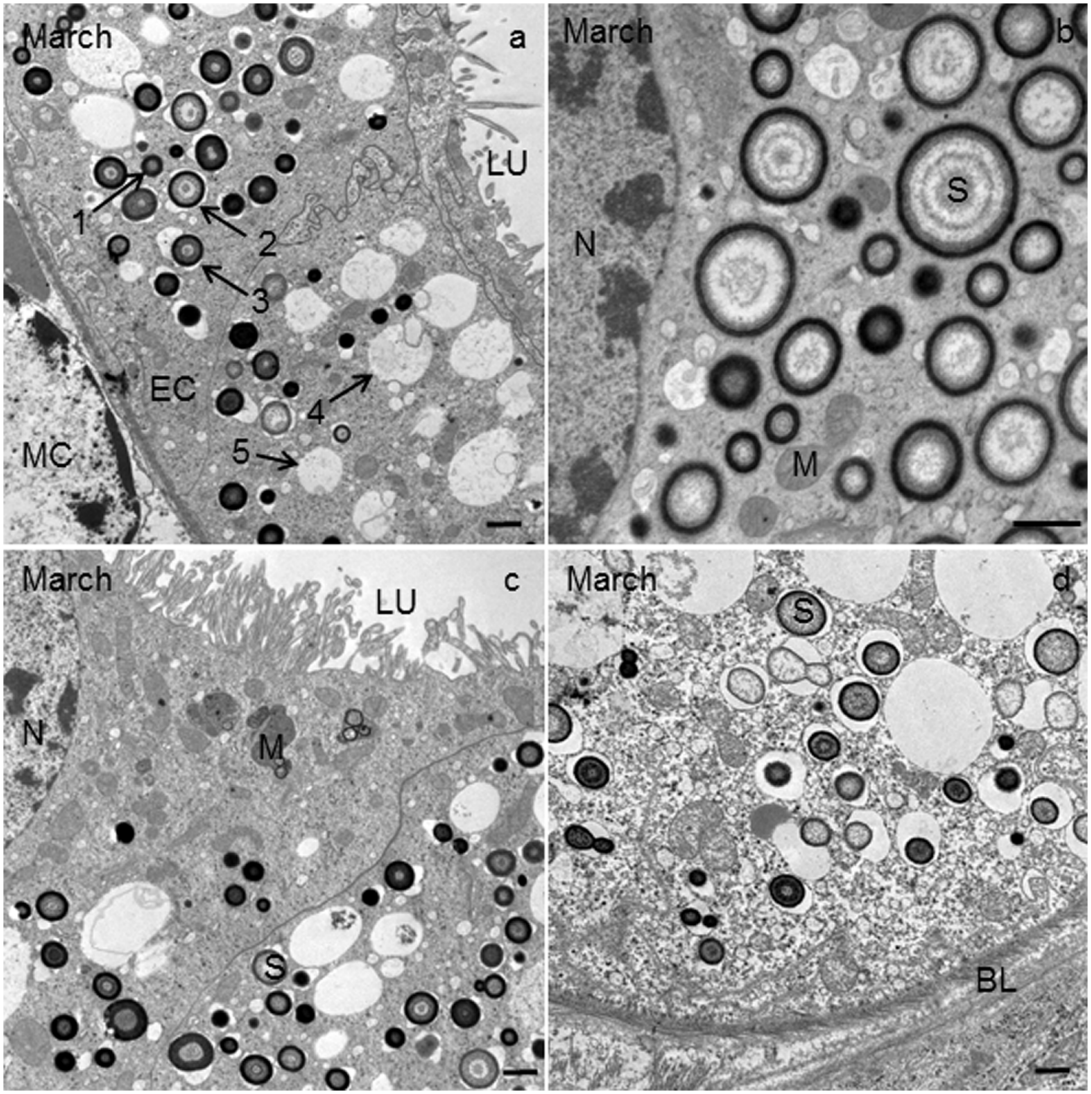
Ultrathin cross-section of the Malpighian tubule (MT) in *T*. *cavicola* at the end of overwintering. **(a)** Epithelial cells (EC) and a muscle cell (MC). Fully-formed spherite in which the dense core is in a contact with the membrane (1). Cases of gradually exploited spherites (2–5). In 2 and 3 the dense spherital cores lost the contact with the membrane. In 4, only one concentric ring of the spherite material and the membrane could be recognized. In 5, the dense core of the spherite is completely exploited; only the membrane is present. LU, lumen of the MT; S, spherite. Scale bar: 2 μm. **(b)** The perinuclear region of the EC with spherites (S). Scale bar: 1 μm. **(c)** Apical part of the EC. LU, lumen of the MT; M, mitochondrium; S, spherite. Scale bar: 2 μm. **(d)** Basal part of the EC and the basal lamina (BL). S, spherite. Scale bar: 2 μm.

The morphology of compartments such as rER, Golgi apparatus, mitochondria apparently did not change in *T*. *cavicola*. The material of many spherites was partly or completely exploited (Fig 7a). Most spherites in the perinuclear cytoplasm contained electron-lucent layers (Fig 7b). Autophagic structures were present in many cells (Table 1). Among these, autophagosomes (Fig 6c and 6d) and the residual bodies (Fig 6d) were most abundant (Fig 6c and 6d). Using IFM, the high abundance of autophagosomes was also visible (Fig 4c).

### Morphometric analysis

**Fig 8:**
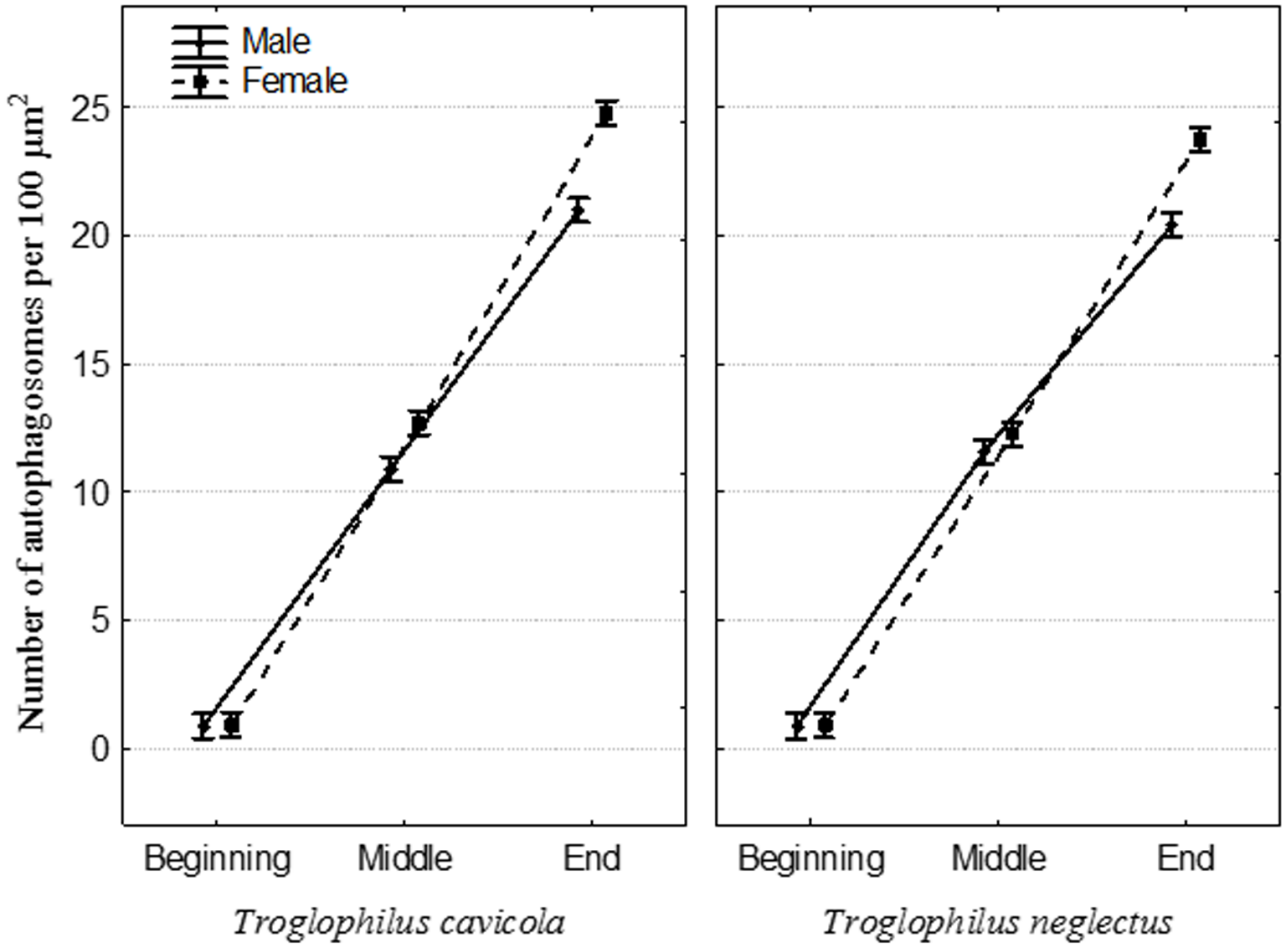
Abundances of autophagosomes per 100 μm^2^ in the epithelial cells of the Malpighian tubules in *Troglophilus cavicola* and *T*. *neglectus* during three time frames of overwintering, as determined using IFM.

Before overwintering, in both sexes in both species the percentage of MT epithelial cells with autophagic structures were low, but these gradually increased until the end of overwintering, as found by TEM (Table 1) and IFM (Table 2, Fig 8). In both sexes of *T*. *cavicola*, the numbers of autophagosomes increased between the beginning and the end of overwintering (Table 2). At the beginning of overwintering, there were no significant differences between sexes in any of the species under investigation, while significant differences appeared in *T*. *cavicola* in the middle of overwintering and at the end of overwintering (Table 2) and in *T*. *neglectus* at the end of overwintering. When males and females were compared separately between species, there were no significant differences in autophagosome abundances in November and January for both sexes (Table 3). A significant difference was found only in females in March, when the abundance of autophagosomes was larger in *T*. *cavicola* than in *T*. *neglectus* (Table 3, Fig 8).

**Table 2:** Descriptive statistics (mean ± StD; min − max) of autophagosome abundances per 100 μm^2^ in the epithelial cells of the Malpighian tubules in *Troglophilus cavicola* and *T*. *neglectus*, and Mann-Whitney U test between the sexes. Significant differences in bold.

Species	Time frame of overwintering	Male	Female	Mann-Whitney U test between sexes,
		$\bar{x}$ ± StD	$\bar{x}$ ± StD	df = 398
		Min—Max	Min—Max	
***T*. *cavicola***	Beginning	0.9 ± 1.0	0.9 ± 1.2	Z = 0.49; p = 0.625
		0–4	0–6	
	Middle	10.9 ± 4.0	12.7 ± 3.5	**Z = 4.66; p < 0.001**
		0–21	3–21	
	End	21.0 ± 5.1	24.8 ± 4.9	**Z = 6.75; p < 0.001**
		6–29	10–37	
***T*. *neglectus***	Beginning	0.9 ± 1.2	0.9 ± 1.3	Z = 0.07; p = 0.944
		0–6	0–7	
	Middle	11.6 ± 3.3	12.3 ± 3.8	Z = 1.38; p < 0.169
		4–22	4–21	
	End	20.4 ± 4.2	23.8 ± 4.4	**Z = 6.95; p < 0.001**
		9–29	14–34	

**Table 3:** Differences in autophagosome abundances per 100 μm^2^ in the epithelial cells between each sex of *Troglophilus cavicola* and *T*. *neglectus* using Mann-Whitney U test. Significant differences in bold.

	Mann-Whitney U test between species	
	df = 398	
Time frame of overwintering	Male	Female
Beginning	Z = 1.03	Z = 0.35
	P = 0.302	P = 0.729
Middle	Z = 1.83	Z = 1.52
	P = 0.068	P = 0.128
End	Z = 1.82	**Z = 2.25**
	P = 0.069	**P = 0.025**

## Discussion

In both cave crickets under study, *T*. *cavicola* and *T*. *neglectus*, the MT epithelial cells were typically shaped and structured, as described in MTs of other insects, e.g. *Schistocerca gregaria* [40], *Acheta domesticus* [41] and *Sarcophaga ruficornis* [42].

Overwintering is a natural starvation period when many metabolic processes occur in cells to maintain the basal organismic metabolism. At the beginning of overwintering, cells are considered to be normally fed and, consequently, characterized by their usual shape and structure [40, 42–44]. In the MT epithelial cells in both cave cricket species and both sexes, the nuclei and compartments such as rER, Golgi apparatus, mitochondria did not show ultrastructural changes during overwintering. Besides, abundant mitochondria in the apical and the perinuclear cytoplasm denote that both are engaged in energy-consuming processes: probably, the apical cell part is mostly engaged in active transport, and the perinuclear part in intensive synthesis in rough and smooth ER [45]. The Golgi apparatus and rER are abundant in the perinuclear part in accordance with their functions [46]. Numerous spherites close to the nucleus reveal that they abundantly support nutrient-consuming processes in this part of the cell.

During overwintering, in both *Troglophilus* species the most conspicuous were gradually mounting structural changes of the spherites and autophagic structures, as it was also observed in the fat body cells of overwintering *T*. *neglectus* [38], and in the cells of the midgut diverticula in overwintering *Gyas annulatus* [47]. In both *Troglophilus* species, the abundance of the spherites did not change during overwintering, but they gradually showed much more electron-lucent material, which reveals their intensive exploitation [48]. A previous study of the chemical elements contained in spherites of *T*. *neglectus*, showed that Mg, Fe, S and Zn were completely exploited during overwintering [21]. Mg and Fe are thought to be used in the respiratory chain in the inner mitochondrial membrane [49]. S is an essential compound of cysteine, methionine and many enzymes which handle acyl-containing biochemical, e.g. the Co-A, and Zn is an important part of many enzymes, including phospholipases involved in the metabolism of lipids [50]. This study showed that gradually, four types of autophagic structures appeared: the phagophores, the autophagosomes, the autolysosomes and the residual bodies. This is the usual order in the self-metabolizing processes within a cell [30, 33].

As in the fat body of *T*. *neglectus* [38], TEM and IFM provided congruent results in the MTs. All these autophagic structures have been found in both the fat body and the MTs of starved *T*. *cavicola* and *T*. *neglectus*, in each case more and more autophagic structures were found during the course of overwintering (see [38] and this study), suggesting that in these species the autophagic processes in the the fat body and the MTs are parallel and have a very similar sequence. The autophagic processes in *T*. *neglectus* are quite comparable with those in *T*. *cavicola*. However, as had been expected, in *T*. *neglectus*, the autophagic cells are more abundant in the fat body [38], which is in accordance with its central metabolic role during overwintering. It is expected that the same will be found in *T*. *cavicola*. Although *T*. *cavicola* show a more glycogen dependent metabolism and *T*. *neglectus* a more lipid dependent one [37], this does not influence the other metabolic processes within the cells of the fat body and the MTs during overwintering.

This study shows that autophagosomes accumulated significantly in the apical and the perinuclear areas of the MT epithelial cells during quiescence in *T*. *neglectus* and during diapause in *T*. *cavicola*. The abundance of the autophagic structures gradually augmented during overwintering. An explanation may be that trafficking to lysosomes may be reduced [33], so autophagosomes are not being degraded. The greater extent of autophagy in diapausing *T*. *cavicola* in comparison to quiescent *T*. *neglectus*, on the one hand, and a greater extent in *T*. *cavicola* females than in males, on the other hand, suggests a higher level of the basal metabolism in *T*. *cavicola*. The most common mode of reproduction in insects is by yolked eggs, when females accumulate large amounts of proteins and lipids [51]. In female *T*. *cavicola* only, at the end of overwintering, abundant ER in the adipocytes prove their biosynthetic activity, which most probably refers to the egg production during diapause (unpublished personal observation; [37]). Therefore the major quantitative differences found in diapausing *T*. *cavicola* females in March are most likely because of oogenesis, as compared to quiescent *T*. *neglectus* females. This is congruent with the expectation that diapause is a more energy- and nutrient-consuming process than quiescence, and that oogenesis is a more energy- and nutrient-consuming process than spermatogenesis.

## Conclusion

In the dormant cave crickets *Troglophilus cavicola* and the closely related *T*. *neglectus*, the main changes in the MT epithelial cells, as well as the fat body cells in both species and sexes are parallel and similar. In these two species, quantitative differences refer to various overwintering strategies, i.e., diapause vs. quiescence. The major quantitative differences affect diapausing *T*. *cavicola* females in March because of oogenesis, as compared to quiescent *T*. *neglectus* females.

## References

[1] Remy P. Observations sur les moeurs de quelques Orthoptères cavernicoles Annales des Sciences Naturelles—Zoologie et Biologie Animale 1931; 14: 263–274.

[2] Chopard L. La Biologie des Orthoptères. P. Lechevalier, Paris, 1983; 541.

[3] Leroy Y. Gryllides et Gryllacrides cavernicoles Annales de Spéléologie 1967; 22: 659–721.

[4] Di Russo C, Sbordoni V. Gryllacridoidea In: Juberthie C, Decu V, editors,Encyclopedia Biospeleologica Vol. II Bucharest: Moulis; 1998 pp. 989–1001.

[5] Sbordoni V, Cobolli M. Insecta: Pterygota In: Gunn GW, editor. Encyclopaedia of Caves and Karst Science. New York, London: Fitzroy Dearbor; 2004 pp. 451–453.

[6] Di Russo C, Rampini M, Landeck I. The cave crickets of northeast Turkey and transCaucasian regions, with descriptions of two new species of the genera *Dolichopoda* and *Troglophilus* (Orthoptera, Rhaphidophoridae). J Orthoptera Res. 2007; 16: 67–76.

[7] Heller GH. Fauna Europea: Rhaphidophoridae. Fauna Europaea 2004; Version 1.1 Available: http://www.faunaeur.org.

[8] Alexiou S, Di Russo C, Rampini M. The family Rhaphidophoridae (Orthoptera) in Greece Parnassiana Archives 2013; 1: 51−58.

[9] Moog O. Die Verbreitung der Höhlenheuschrecken *Troglophilus cavicola* KOLLAR und *T* *neglectus* KRAUSS in Österreich (Orthoptera: Rhaphidophoridae). –Sitzber. Österr. Akad. Wiss., Mathem.-naturw. Kl., 1982; Abt. I 191 (5–10), 185–207.

[10] Novak T, Kuštor V. On *Troglophilus* (Rhaphidophoridae, Saltatoria) from North Slovenia (YU). Mémoires de Biospéologie 1983; 10: 127−137.

[11] Pehani Š, Virant-Doberlet M, Jeram S. The life cycle of the cave cricket *Troglophilus neglectus* Krauss with a note on *T*. *cavicola* Kollar (Orthoptera: Rhaphidophoridae). The Entomologist 1997; 116: 224–238.

[12] Ketmaier V, Cobolli M, De Matthaeis E, Rampini M. Biochemical systematics and patterns of genetic divergence between the *Troglophilus* species of Crete and Rhodos (Orthoptera, Rhaphidophoridae). Belg J Zool. 2000; 130 (1): 49–53.

[13] Christian E. Höhlenheuschrecken—Zum Jubiläum einer Wortschöpfung. Die Höhle 2008; 59: 48–58.

[14] Karaman I, Hammouti N, Pavićević D, Kiefer A, Horvatović M, Seitz A. The genus *Troglophilus* Krauss, 1879 (Orthoptera: Rhaphidophoridae) in the west Balkans. Zool J Linn Soc. 2011; 163: 1035–1063.

[15] Taylan S,M, Di Russo C, Cobolli M, Rampini M. New species from the genus *Troglophilus* Krauss, 1879 (Orthoptera, Rhaphidophoridae) from Western and Southern Anatolian caves, Turkey. Zootaxa 2012; 3597: 33–40.

[16] Taylan SM, Di Russo C, Rampini M, Ketmaier V. Molecular systematics of the genus *Troglophilus* (Rhaphidophoridae, Orthoptera) in Turkey: mitochondrial 16S rDNA evidences. ZooKeys. 2013; 257: 33–46. 10.3897/zookeys.257.4133 23653493 PMC3591738

[17] Kaya S, Boztepe Z, Çiplak B. Phylogeography of *Troglophilus* (Orthoptera: Troglophilinae) based on Anatolian members of the genus: radiation of an old lineage following the Messinian. Biol J Linn Soc. 2013; 108: 335–348.

[18] Novak T, Janžekovič F, Lipovšek S. Contribution of non-troglobiotic terrestrial invertebrates to carbon input in hypogean habitats. Acta Carsologica 2013; 42: 301−309.

[19] Hahn DA, Denlinger DL. Energetics of insect diapause. Annu Rev Entomol. 2011; 56: 103−121. 10.1146/annurev-ento-112408-085436 20690828

[20] Kögler K. Aktivitätsverhalten und Orientierung in Temperaturgradienten von *Troglophilus cavicola* Kollar im Jahreslauf. Dissertation, University of Graz 1983.

[21] Lipovšek S, Letofsky-Papst I, Novak T, Hofer F, Pabst MA.Structure of the Malpighian tubule cells and annual changes in the structure and chemical composition of their spherites in the cave cricket *Troglophilus neglectus* Krauss, 1878 (Rhaphidophoridae, Saltatoria). Arthropod Struct Dev. 2009; 38: 315–327. 10.1016/j.asd.2009.02.001 19303052

[22] Bradley TJ. The excretory system: structure and physiology In: Kerkut GA, Gilbert LI, editors. Comprehensive Insect Physiology, Biochemistry and Pharmacology, New York: Pergamon Press;, 1985 pp. 421–465.

[23] Dow JAT. Insights into the Malpighian tubule from functional genomics. J Exp Biol. 2009; 212: 435–445. 10.1242/jeb.024224 19151219

[24] Beyenbach KW, Skaer H, Dow JAT. The development, molecular, and transport Biology of Malpighian tubules. Annu Rev Entomol. 2010; 55: 351–374. 10.1146/annurev-ento-112408-085512 19961332

[25] Bradley TJ. Active transport in insect recta. J Exp Biol 2008; 211: 835−836. 10.1242/jeb.009589 18310107

[26] Martoja R, Ballan-Dufrançais C. The ultrastructure of the digestive and excretory organs In: King RC, Akai H (Eds.), Insect Ultrastructure. Plenum, New York, 1984:199–268.

[27] He C, Klionsky DJ. Regulation mechanisms and signaling pathways of autophagy. Annu Rev Genet. 2009; 43: 67–93. 10.1146/annurev-genet-102808-114910 19653858 PMC2831538

[28] Russel RC, Yuan H-X, Guan K-L. Autophagy regulation by nutrient signaling. Cell Res. 2014; 24: 42–57. 10.1038/cr.2013.166 24343578 PMC3879708

[29] Mizushima N, Yamamoto A, Matsui M, Yoshimori T, Ohsumi Y. In vivo analysis of autophagy in response to nutrient starvation using transgenic mice expressing a fluorescent autophagosome marker. Mol Biol Cell. 2004; 15: 1101–1111. 14699058 10.1091/mbc.E03-09-0704 PMC363084

[30] Kristensen AR, Schandorff S, Høyer-Hansen M, Overbeck Nielsen M, Jäättelä M, Dengjel J et al Ordered organelle degradation during starvation-induced autophagy. Mol Cell Proteomics. 2008; 7(12): 2419–2428. 10.1074/mcp.M800184-MCP200 18687634

[31] Barth JM, Szabad J, Hafen E, Kohler K. Autophagy in *Drosophila* ovaries is induced by starvation and is required for oogenesis. Cell Death Differ. 2011; 18: 915–924. 10.1038/cdd.2010.157 21151027 PMC3131947

[32] Khoa DB, Takeda M. Expression of autophagy 8 (Atg8) and its role in the midgut and other organs of the greater wax moth, *Galleria mellonella*, during metamorphic remodelling and under starvation. Insect Mol Biol. 2012; 21(5): 473–487. 10.1111/j.1365-2583.2012.01152.x 22830988

[33] Klionsky DJ, Abdalla FC, Abeliovich H, Abraham RT, Acevedo-Arozena A, Adeli K. Guidelines for the use and interpretation of assays for monitoring autophagy. Autophagy. 2012; 8(4): 445–544. 22966490 10.4161/auto.19496 PMC3404883

[34] Arrese EL, Soulages JL. Insect fat body: energy, metabolism, and regulation. Annu Rev Entomol. 2010; 55: 207−225. 10.1146/annurev-ento-112408-085356 19725772 PMC3075550

[35] Panabecker T. Physiology of the Malpighian tubule. Annu Rev Entomol. 1995; 40: 493−510.

[36] Pacheco CA, Alevi KCC, Ravazi A, de Azeredo Oliveira MTV. Review: Malpighian tubule, an essential organ for insects. Entomol Ornithol Herpetol. 2014; 1000122. 3:122 10.4172/2161-0983.1000122

[37] Lipovšek S, Novak T, Janžekovič F, Pabst MA. Role of the fat body in the cave crickets *Troglophilus cavicola* and *Troglophilus neglectus* (Rhaphidophoridae, Saltatoria during overwintering. Arthropod Struct Dev. 2011; 40(1): 54–63. 10.1016/j.asd.2010.09.002 20868768

[38] Lipovšek S, Novak T. Autophagy in the fat body cells of the cave cricket *Troglophilus neglectus* Krauss, 1878 (Rhaphidophoridae, Saltatoria) during overwintering. Protoplasma 2015; 10.1007/s00709-015-0824-3 25956501

[39] Eskelinen E-L. Fine structure of the autophagosome In: Deretic V, editor. Methods in Molecular Biology, Vol. 445. Autophagosome and phagosome. New York: Humana Press 2008; pp. 11–28. 10.1007/978-1-59745-157-4_2 18425441

[40] Garrett MA, Bradley TJ, Meredith JE, Phillips JE.Ultrastructure of the Malpighian tubules of *Schistocerca gregaria*. Journal of morphology 1988; 195: 313–325. 29898572 10.1002/jmor.1051950306

[41] Hazelton SR, Felgenhauer BE, Spring JH. Ultrastructural changes in the Malpighian tubules of the house cricket, *Acheta domesticus*, at the onset of diuresis: a time study. Journal of morphology 2001; 247: 80–92. 11124687 10.1002/1097-4687(200101)247:1<80::AID-JMOR1004>3.0.CO;2-X

[42] Pal R, Kumar K. Malpighian tubules of adult flesh fly, *Sarcophaga ruficornis* Fab. (Diptera: Sarcophagidae): An ultrastructural study. Tissue Cell 2013; 45: 312–317. 10.1016/j.tice.2013.04.002 23664310

[43] Yea SY, Yu CH. Morphology of the Malpighian tubules in the German cockroach, *Blattella germanica* L. Bulletin of the Institute of Basic Science, Inha University 1992; 13: 97–105.

[44] Kalender Y, Kalender S, Candan S. Fine structure of Malpighian tubules in the *Agrotis segetum* (Lepidoptera: Noctuidae) pupae. Acta Zoologica Bulgarica 2002; 54: 87–96.

[45] Alberts B, Johnson A, Lewis J, Morgan D, Raff M, Roberts K et al Intracellular Compartments and Protein Sorting In: Alberts B. et al (Eds). Molecular Biology of the Cell, 6^th^ Edition, Garland Science, Taylor & Francis Group, New York, 2014.

[46] Cooper GM, Hausman RE. Protein Synthesis, Processing, and Regulation In: Cooper G. M. and Hausman R. E. (Eds.) The Cell. A Molecular Approach. ASM Press, Washington D.C., 2009; 309–352.

[47] Lipovšek S, Janžekovič F, Novak T. Autophagic activity in the midgut gland of the overwintering harvestmen *Gyas annulatus* (Phalangiidae, Opiliones). Arthropod Struct Dev. 2014; 43, 493–500. 10.1016/j.asd.2014.06.001 24929120

[48] Pigino G, Migliorini M, Paccagnini E, Bernini F, Leonzio C. Fine structure of the midgut and Malpighian papillae in *Campodea (Monocampa) quilisi Silvestri*, 1932 (Hexapoda, Diplura) with special reference to the metal composition and physiological significance of midgut intracellular electron-dense granules. Tissue Cell. 2005; 37: 223–232. 15936358 10.1016/j.tice.2005.02.001

[49] Alberts B, Johnson A, Lewis J, Raff M, Roberts K, Walter P et al Energy Conversion: Mitochondria and Chloroplasts In: Alberts B, editor. Molecular Biology of the Cell. New York, Abington: Garland Science, Taylor & Francis Group; 2008 pp. 813–878.

[50] Alberts B, Bray D, Hopkin K, Johnson A, Lewis J, Raff M et al Chemical Components of Cells In: Alberts B, editor. Essential Cell Biology. New York, Abingdon: Garland Science, Taylor & Francis Group;, 2004 pp. 39–81.

[51] Wheeler DE. Reproduction, female In: Resh V H, Cardé RT, editors. Encyclopaedia of insects. Amsterdam: Academic Press; 2003 pp. 991−993.

